# Effect of "no added salt diet" on blood pressure control and 24 hour urinary sodium excretion in mild to moderate hypertension

**DOI:** 10.1186/1471-2261-7-34

**Published:** 2007-11-06

**Authors:** Javad Kojuri, Rahim Rahimi

**Affiliations:** 1 Shiraz University of Medical Sciences, Shiraz, Iran

## Abstract

**Background:**

The incidence of Hypertension as a major cardiovascular threat is increasing. The best known diet for hypertensives is 'no added salt diet'.

In this study we evaluated the effect of 'no added salt diet' on a hypertensive population with high dietary sodium intake by measuring 24 hour urinary sodium excretion.

**Methods:**

In this single center randomized study 80 patients (60 cases and 20 controls) not on any drug therapy for hypertension with mild to moderate hypertension were enrolled. 24 hour holter monitoring of BP and 24 hour urinary sodium excretion were measured before and after 6 weeks of 'no added salt diet'.

**Results:**

There was no statistically significant difference between age, weight, sex, Hyperlipidemia, family history of hypertension, mean systolic and diastolic BP during the day and at night and mean urinary sodium excretion in 24 hour urine of case and control groups. Seventy eight percent of all patients had moderate to high salt intake.

After 6 week of 'no added salt diet' systolic and diastolic BP significantly decreased during the day (mean decrease: 12.1/6.8 mmhg) and at night (mean decrease: 11.1/5.9 mmhg) which is statistically significant in comparison to control group (P 0.001 and 0.01).

Urinary sodium excretion of 24 hour urine decreased by 37.1 meq/d ± 39,67 mg/dl in case group which is statistically significant in comparison to control group (p: 0.001).

Only 36% of the patients, after no added salt diet, reached the pretreatment goal of 24 hour urinary sodium excretion of below 100 meq/dl (P:0.001).

**Conclusion:**

Despite modest effect on dietary sodium restriction, no added salt diet significantly decreased systolic and diastolic BP and so it should be advised to every hypertensive patient.

**Trial Registration:**

Clinicaltrial.govnumber NCT00491881

## Background

Hypertension is still one of the most common causes of cardiac, renal and cerebrovascular diseases [1]. Hypertension is a silent risk factor and only one third of hypertensive patients are diagnosed and of these only one third are treated properly (BP < 120/80) [2].

The incidence of hypertension, regarding modern sedentary life style and population aging, is increasing; however due to asymptomatic and chronic nature of the disease which mandates life long drug therapy, its control is less satisfactory [3].

Hypertension is strongly affected by genetic and environmental factors, among which, low salt diet is the most applicable and important modifiable factor [4].

Strict dietary sodium control is no longer recommended owing to the fact that only 30% of patients are salt sensitive and the use of diuretics can effectively decrease body sodium reserve [5].

Nowadays the best applicable diet for hypertensives is DASH diet (Dietary Approach to Stop Hypertension) and 'no added salt diet' approach [6], which can effectively decrease systolic and diastolic pressures by 11.4 and 5.5 mmHg respectively [1,6].

Simple 'no added salt diet' (not adding salt on table and not using salty foods) can effectively decrease systolic and diastolic pressures by 5.5 and 3 mmHg [7].

'No added salt diet' if applied properly, should decrease salt intake to below 5 gram and sodium chloride to below 2.4 gram in 24 hours and this would decrease urinary sodium excretion to below 100 meq/dl in 24 hour urine [4,8,9].

In this study, we evaluated the effect of no added salt diet on a hypertensive population with high dietary sodium intake by measuring 24 hour urinary sodium excretion as an index of the effectiveness of this regimen on restriction of sodium intake.

## Methods

In a single center randomized study, 80 patients with mild to moderate hypertension, not on any antihypertensive drugs, were enrolled.

Twenty four hour holter monitoring of blood pressure was recorded with Davinsa device from right arm, for 24 hours from 8 AM to 8 AM next day. Patients were told to follow their daily activities and not using any medications for these 24 hours. Twenty four hour urine of all cases was collected from 8 AM to 8 AM next day, and patients were instructed to collect all of their urines even at night. Twenty four hour urinary sodium and creatinine excretion were measured in all patients. Coronary risk factors, weight, and routine dietary intake of all patients were recorded. *Low salt diet was defined as sodium intake below 3 gr/day, moderate salt intake as sodium intake of 3–7 gr/day, and high salt diet as more than 7 gr/day of sodium intake *[10]. Based on this classification and qualitative assessment of weekly diet intake, patients were divided to three classes of low, medium, and high sodium intake. Those who took salty food and bread with salty appetizers (based on sodium content of each food on their labels or instructions) were classified as high sodium users. Patients were selected randomly from outpatient clinic of Shiraz University from among those who referred with mild to moderate hypertension, and were not on any antihypertension medications. Control group were also selected randomly from among healthy subjects referring for annual check ups after matching for age & sex. No added salt/DASH diet was completely explained to all cases. After six weeks, weight, 24 hour holter of blood pressure and 24 hour urinary sodium excretion were measured again in both groups under the same conditions.

This study was approved by vise chancellor for research and ethics committee of Shiraz University of medical sciences and all patients filled the written consent before enrollment.

Data are expressed as mean ± SD. Mann-Whitney *U*-test was used to compare differences between the cases and controls. A value of *P *< 0.05 was considered statistically significant.

## Results

There was no statistically significant difference for age, weight, sex, hyperlipidemia and family history of hypertension between the two groups (Table 1). Mean systolic and diastolic blood pressures during the day and at night and the mean urinary sodium excretion in 24 hour urine were not also statistically different between the both groups (Table 1). Smoking was more common in the control group (25% vs. 5%, P: 0.011, DF: 1) (Table 1).

**Table 1:** Descriptive data of case and control groups

	**Case**	**Control**	**P Value**
Age (year)	48.7 ± 11.1	46.05 ± 13.173	0.41
Weight (kilogram)	69.47 ± 10.39	70.85 ± 10.15	0.62
Systolic BP (day)	147.1 ± 12.7	141.2 ± 10.2	0.06
Systolic BP(night)	136.7 ± 9.2	133.3 ± 10.9	0.173
Urinary Na excretion in 24 hour urine (meq/dl)	132(35–200)	216(210–274)	0.42
Urinary Na per creatinine excretion in 24 hours urine (meq/mg/dl)	1.44 ± 0.63	1.52 ± 0.68	0.46
Hyperlipidemia*	31 (52%)	7 (35%)	0.175
Family history**	11 (18%)	7 (35%)	0.07
Smoking***	3 (5%)	5 (25%)	0.011
Male sex	30 (50%)	10 (50%)	1.0

Data are expressed as Mean ± SD, except of urinary sodium excretion which is expressed as median and its range.

*Defined as history of triglyceride more than200 mg/dl and total cholesterol more than 200 mg/dl.

**History of hypertension (BP more than 140/90) in fist degree relatives.

*** Smoking recent smoking of even a cigarette per day

Patients were divided to three groups of low, medium and high salt dietary intake based on qualitative evaluation of their routine weekly food intake. Twenty one percent of patients used low salt, 52% used medium, and 26% used high sodium diet, this trend was almost the same for both the case and the control group (P: 0.417, df: 2, SF: 1.748).

The mean systolic and diastolic pressures during the day decreased by 12.1 and 6.8 mmHg and the mean systolic and diastolic pressures at night decreased by 11.1 and 5.9 mmHg respectively in the case group which was statistically significant in comparison to control group (P:0.001, df:78, SF:2.38 for systolic and 0.01 for diastolic pressures) (Table 2).

**Table 2:** Evaluation of difference of pre-therapy and post-therapy data in case and control groups

**Difference of pre therapy & post therapy data**	**Case**	**Control**	**P value**
Systolic BP (day)	12.1 ± 9.2	-4.9 ± 6.6	0.001
Systolic BP(night)	11.13 ± 9.8	-1.3 ± 3.9	0.001
Diastolic BP(day)	6.8 ± 7.3	-2.4 ± 3.3	0.01
Diastolic BP (night)	5.9 ± 4.8	-1.1 ± 3.7	0.01
Urinary Na excretion in 24 Hr urine (meq/dl)	42(-100 to 110)	-4(-69 to +23)	0.001

Data are expressed as Mean ± SD, except of urinary sodium excretion which is expressed as median and range.

Urinary sodium excretion in 24 hour urine decreased by 37.1 meq/dl (37.1 ± 39.67 meq/dl) in cases and increased by 10.7 ± 26.07 meq/dl in the control group which was statistically significant (P: 0.001 df: 78, SE: 9.5) (Table 2). For better comparison urinary sodium excretion was compared as median and range between two groups, pre intervention urinary sodium excretion median was 132 meq/dl (35–200) for cases and 216 meq/dl (210–274) for control group(p:0.42), and post intervention urinary sodium excretion was 110 meq/dl (50–174) for cases and 200 meq/dl (178–279) for control group (p:0.01). Thirty five percent of patients reached the urinary sodium excretion level less than 100 meq/dl in 24 hours after 6 weeks of taking the diet (prior to diet 8%, after diet 35%, P:0.0001) (Table 2). Decrease in systolic pressure at night to more than 15% (night dipping of pressure) was not statistically significantly increased after taking 6 weeks of 'no added salt diet'(P:0.241, SE:2.05).

## Discussion

Despite significant effect on systolic and diastolic pressures during the day and at night [1,6,7], 'no added salt diet' could only decrease the urinary sodium excretion of 35% of patients to the pretreatment determined level of less than 100 meq/dl in 24 hours [9]. This may be due to high intake of salt in the general population diet. Our culture dictates use of tasty and salty food and bread which could not be eliminated from hypertensive patient's diet and consequently would increase urinary sodium excretion even after 6 weeks of strict attachment to 'no added salt diet'. This could be noticed by high urinary sodium excretion in 24 hour urine of patients before enrollment (mean urinary sodium excretion of 163.2 eqg ± 48.99) and this fact that 78% of patients received medium to large amount of dietary sodium intake based on qualitative evaluation [10]. This could be rationally explained the modest effect of 'no added salt diet' in general population, because many sources of salt intake, for example bread, convenience, and fast foods which are rich sources of sodium intake, could not be eliminated from the diet as much as the patients and those who take care of them tried.

However, despite modest effect on sodium restriction, 'no added salt diet' can effectively decrease both systolic and diastolic blood pressures during the day and at night time[1,6,7], which means even modest decrease in salt intake by simple advice, limit the use of salty foods and do not use salt on table, could significantly decrease pressures both in men and women (mean day systolic pressure decrease of 9.8 mmhg in males and 5.9 mmhg in females which was not statistically significant in male and female population) (P: 0.138, SF: 2.6).

There is much evidence that a reduction in dietary salt intake lowers blood pressure in hypertensive individuals. However, few have looked at the effect of 'no added salt diet' and modest salt intake on total restriction of sodium intake with especial attention to very exact surrogate of urinary sodium excretion. Our study demonstrates that a modest reduction in salt intake from regular level of 10 – 12 g per day to the recommended level of 5 – 6 g per day lowers blood pressure by 12.1/6.8 mmHg during the day and 11.1/5.9 mmhg at night. This decrease in systolic and diastolic pressures with salt reduction in 'no added salt diet' would be predicted to reduce stroke by more than one third, ischemic heart disease by one quarter, and heart failure by just over one quarter in hypertensive population [11].

In our study, the fall in systolic blood pressure with salt reduction is greater than the fall in diastolic pressure; therefore, there is a fall in pulse pressure. Pulse pressure is a surrogate marker of artery stiffness, which has been suggested to be an independent risk factor for cardiovascular disease [12]. So our study suggests that 'no added salt diet' may improve arterial distensibility, too.

These results provide strong support for universal salt reduction in all hypertensive individuals irrespective of modest effect of 'no added salt diet'.

Hypertensive patients whose blood pressures drop at night, so-called night dippers, have less cardiovascular complications [13]. In our study night dipping rate did not increase after 6 weeks of 'no added salt diet', this suggests that night dipping may be a genetic factor and could not be enhanced by 'no added salt diet' [14]. Further studies are needed to evaluate the effect of diet on night dipping of blood pressure.

## Conclusion

Concerning its ease of application and significant effect, 'no added salt diet 'can be recommended to all patients with hypertension. However it should be notified that the effect of 'no added salt diet' on salt intake restriction is moderate at most.

## Study limitation

1) Limited number of study groups which mandates larger scale, population based studies to evaluate the effect of 'no added salt diet'.

2) Absence of quantitative evaluation of dietary sodium intake

## Competing interests

The author(s) declare that they have no competing interests.

## Authors' contributions

JK carried out design of the study, data collection, writing the article, and statistical analysis. RR carried out data collection & preliminary report. All authors read and approved the final manuscript.

## Pre-publication history

The pre-publication history for this paper can be accessed here:


